# Contrast Media Viscosity versus Osmolality in Kidney Injury: Lessons from Animal Studies

**DOI:** 10.1155/2014/358136

**Published:** 2014-02-23

**Authors:** Erdmann Seeliger, Diana C. Lenhard, Pontus B. Persson

**Affiliations:** Institute of Physiology and Center for Cardiovascular Research, Charité-University Medicine Berlin, Campus Mitte, Hessische Straße 3-4, 10115 Berlin, Germany

## Abstract

Iodinated contrast media (CM) can induce acute kidney injury (AKI). CM share common iodine-related cytotoxic features but differ considerably with regard to osmolality and viscosity. Meta-analyses of clinical trials generally failed to reveal renal safety differences of modern CM with regard to these physicochemical properties. While most trials' reliance on serum creatinine as outcome measure contributes to this lack of clinical evidence, it largely relies on the nature of prospective clinical trials: effective prophylaxis by ample hydration must be employed. In everyday life, patients are often not well hydrated; here we lack clinical data. However, preclinical studies that directly measured glomerular filtration rate, intrarenal perfusion and oxygenation, and various markers of AKI have shown that the viscosity of CM is of vast importance. In the renal tubules, CM become enriched, as water is reabsorbed, but CM are not. In consequence, tubular fluid viscosity increases exponentially. This hinders glomerular filtration and tubular flow and, thereby, prolongs intrarenal retention of cytotoxic CM. Renal cells become injured, which triggers hypoperfusion and hypoxia, finally leading to AKI. Comparisons between modern CM reveal that moderately elevated osmolality has a renoprotective effect, in particular, in the dehydrated state, because it prevents excessive tubular fluid viscosity.

## 1. Introduction

Iodinated X-ray contrast media (CM) are widely used in diagnostic and therapeutic procedures such as percutaneous cardiac and arterial interventions and contrast-enhanced computed tomography. In general, today's CM are very well tolerated. However, CM can cause acute kidney injury (AKI), in particular, patients with preexisting renal impairment, endothelial dysfunction, and/or diabetes and patients who are not sufficiently prehydrated are at risk. As the numbers of diagnostic and therapeutic procedures are steadily increasing, CM-induced AKI (CIAKI) has become the third leading cause for iatrogenic AKI. Fortunately, CIAKI is a transient disorder in the majority of patients, however, CIAKI can also have severe consequences: Patients with CIAKI suffer from an increased rate of in-hospital complications including an increased mortality rate and may become predisposed to long-term loss of kidney function [1–6].

All classes of CM share certain properties relevant for CIAKI such as some cytotoxicity. However, they vary considerably with regard to osmolality and viscosity, and there is a standing debate on which of these physicochemical properties determines renal safety. One reason for this uncertainty is the lack of a clinical marker for AKI that would be both reliable and practicable. Meanwhile, preclinical studies have helped fill in gaps. In particular, animal experiments have been instrumental in elucidating the pathophysiological mechanisms that rely on CM viscosity and osmolality. The optimum physicochemical properties of CM to avoid CIAKI will be the focus of this review.

## 2. Why Preclinical Studies Could Answer Questions That Clinical Trials Could Not

CIAKI is broadly defined as a decrease in glomerular filtration rate (GFR) within 2 to 3 days following the intravascular administration of CM in the absence of an alternative aetiology [7–9]. For several reasons, this definition is not an ideal one [7–11]. A decrease in GFR can *per se* not attest to kidney injury; it rather indicates that one partial function of the kidney, namely, filtration, is disturbed. Second, in clinical practice, CIAKI is diagnosed by an increase in the surrogate marker for GFR, serum creatinine (SCrea), because direct measurements of GFR by clearance methods require tedious urine collection. Unfortunately, SCrea is a poor marker of GFR: its sensitivity is very low with regard to both the degree and the time course of GFR changes. Due to the exponential relationship between SCrea and GFR, SCrea is very insensitive in patients with preexisting normal GFR [3, 7, 10, 12]. Day-to-day variations in SCrea observed in patients in whom no cause for rapid GFR changes could be identified indicate that SCrea is also insensitive at reduced preexisting GFR [13, 14]. Moreover, due to the kinetics of creatinine distribution among the body fluid compartments, SCrea is notoriously insensitive to rapid GFR changes such as the immediate GFR drop induced by CM: creatinine accumulation may take days, before SCrea increase fulfils diagnostic criteria [7, 10–12, 15, 16]. Reflecting these drawbacks, CIAKI is currently diagnosed when a certain absolute (e.g., 44 or 88 *μ*mol/L) or relative (e.g., 25 or 50%) increase in SCrea is observed within 48 or 72 hours after CM, whereby the optimum margins are still under debate [3, 8–11]. This diagnostic delay poses severe problems with regard to clinical practice, the most obvious being that CIAKI will often go undetected in outpatients [17].

Besides the consequences for patient care, the poor performance of SCrea to reflect changes in GFR has greatly hampered advances in knowledge. The vast majority of clinical trials have been relying on SCrea as the sole end point. Often, the incidence of CIAKI cannot be compared among trials because of the different absolute or percentage margins in SCrea increase and/or the different timing and frequency of post-CM SCrea measurements (e.g., 24 versus 72 hours) [8, 10, 18–21]. Thus, there is general agreement that many important questions, for example, regarding the incidence of CIAKI following contrast-enhanced CT, regarding specific prophylactic strategies or comparisons among CM, are not yet answered unequivocally, because clinical trials, with few exceptions, relied on SCrea as outcome measure [7, 10, 17, 21–28]. Utilization of robust outcome measures such as requirement of dialysis and, if their reliability is convincingly proven, of new serum and urinary markers of renal injury will certainly improve the power of evidence in future clinical trials [11, 29, 30].

Animal studies that made use of clearance methods to directly measure GFR and that assessed various direct and indirect markers of renal injury and functions helped fill in gaps. Thus, experiments run under highly standardised conditions provided direct comparisons between classes of CM and between specific prophylactic strategies. Moreover, *in vitro* and *in vivo* studies elucidated pathophysiological mechanisms of CIAKI so that it became possible in recent years to shape a quite uniform scheme of CIAKI pathophysiology [7]. Mechanisms underlying CIAKI include cytotoxic effects, paracrine factors that affect renal hemodynamics, altered rheological properties that perturb renal hemodynamics and tubulodynamics, and tissue hypoxia. These mechanisms act in concert, yet the individual mechanisms' importance varies with the classes of CM used, with the subject's hydration/volume status, and—in patients—with the degree of preexisting individual risk factors [7]. One factor that has a major impact on tubulodynamics is fluid viscosity. Fluid viscosity is a measure of the fluid's resistance to flow due to friction between neighboring parcels that are moved at different velocities. In the context of CM, the dynamic viscosity (usually given in millipascal second (mPa s)) and the kinematic viscosity (usually given in square millimetre per second (mm^2^/s)) are most relevant. A fluid's kinematic viscosity (which is typically measured by Ubbelohde-type viscometers that make use of gravity to move the fluid within a tube) is converted to its dynamic viscosity by dividing it by the fluid's density. The classes of CM vary considerably with regard to viscosity and osmolality, and both high viscosity and high osmolality have been implicated to play pivotal roles in the pathophysiology of CIAKI.

## 3. Why the Current Labelling of CM Classes Relies on Their Osmolalities

CM for intravascular use are tri-iodinated benzene derivatives. Because their radio-opacity relies on iodine, solutions with high iodine concentration (usually 250–400 mg I/mL solution) are required. This is achieved by high molar concentrations of benzene derivatives. The molar concentration of the CM solution is a major determinant of both its osmolality and its viscosity. However, whereas the osmolality of a given CM solution increases only linearly with the molar concentration, viscosity increases exponentially [31]. The pioneer class of CM comprised solutions of monomers (iothalamate, diatrizoate) at high molar concentrations. These compounds are ionic, which additionally increase the solutions' osmolalities, so that their osmolalities are exceedingly high (~1000–2500 mosmol/kg H_2_O) as compared to blood plasma (~290 mosmol/kg H_2_O) [32]. This class of CM was termed high-osmolar CM (HOCM) and was found to be associated with a considerable risk for CIAKI [1, 32, 33]. As a consequence, CM with lower osmolalities were developed: By forming either ionic dimers (ioxaglate) or nonionic monomers (iopromide, iopamidol, iohexol, ioversol, iomeprol, etc.); osmolalities of ~400–800 mosmol/kg H_2_O were achieved [32]. Although their osmolalities are actually still higher than these of plasma, these compounds are referred to as low-osmolar CM (LOCM). The realisation that LOCM were associated with a marked lower CIAKI incidence than HOCM, particularly in at-risk patients, had two consequences [32, 33]. First, HOCM are virtually no longer in clinical use in Western Europe and the USA. Second, the next goal in CM development seemed obvious: further reduction in osmolality. By creating nonionic dimeric compounds (iodixanol, iotrolan, and iosimenol), iso-osmolar CM (IOCM) were developed [32, 33]. Pure IOCM solutions are actually hypoosmolar; electrolytes are added to the clinically used solution to reach plasma osmolality [34]. The low osmolality achieved with the IOCM came at the price of considerably increased viscosity. At comparable iodine concentration and, thus, comparable X-ray attenuation, nonionic dimer IOCM have about twice the viscosity of nonionic monomer LOCM [32, 35]. The higher viscosity of nonionic dimer IOCM probably relies on number of the compounds' features including the molecules' shape and the flexibility of the bridge between the two benzene nuclei that may lead to their superposition [36–38]. It must be noted, however, that viscosities of all CM classes are markedly higher than those of plasma, ranging from 2.5-fold in HOCM to 11fold in IOCM [32]. Both physicochemical properties of CM, osmolality, and viscosity have majors impact on CM handling in the renal tubules and, thus, their potential to harm.

## 4. Why CM Become Concentrated in the Kidney

**Figure 1:**
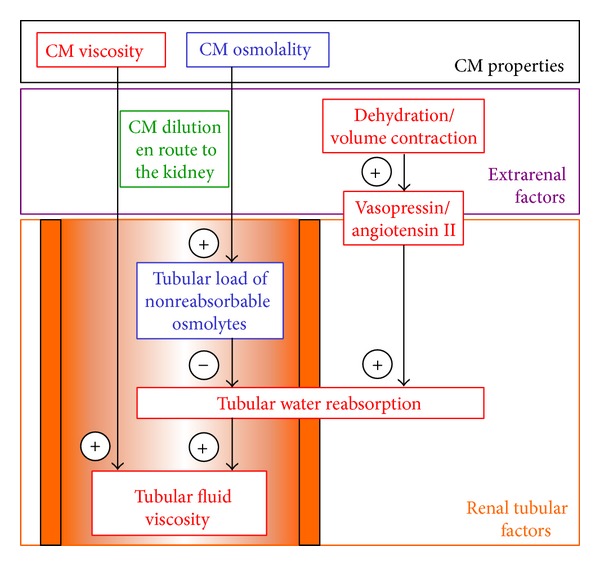
Simplified scheme summarizing major factors/mechanisms that influence tubular fluid viscosity following CM administration. For detailed explanations see text.

CM administered intravascularly are considerably diluted before they reach the kidney, and more so upon intravenous administration such as for contrast-enhanced CT than upon intraarterial administration such as for renovasography and left ventriculography. The different degree of dilution may be a main reason behind the apparent i.v. versus i.a. difference in CIAKI incidence [26–28]. CM dilution en route to the kidney, of course, reduces their viscosity and osmolality (see schematic Figure 1 for factors/mechanisms that influence tubular fluid viscosity following CM administration). Like all other osmolytes small enough to pass the renal glomerular filter, CM are freely filtered so that their concentration in primary urine equals that of the blood plasma entering the kidney. Whereas a multitude of other small osmolytes are reabsorbed by specific tubular transport mechanisms, there are no such transporters for CM. Because the vast majority of the filtered water is reabsorbed along the length of the tubule and CM are not, CM become considerably concentrated en route through the tubules. This results in a progressive increase in tubular fluid osmolality and, due to the exponential concentration-viscosity relationship, an overproportional increase in tubular fluid viscosity. Water reabsorption along the tubules is driven by osmotic gradients between the tubules' lumen and renal interstitial fluid. The renal medulla is unique in that its osmolality is higher than that of all other tissues. In humans, osmotic pressure achieves up to 600 mosmol/kg H_2_O in the outer medulla; in the inner medulla and, thus, also in the long loops of Henle and the inner medullary collecting ducts, it reaches up to 1200 mosmol/kg H_2_O.

**Figure 2:**
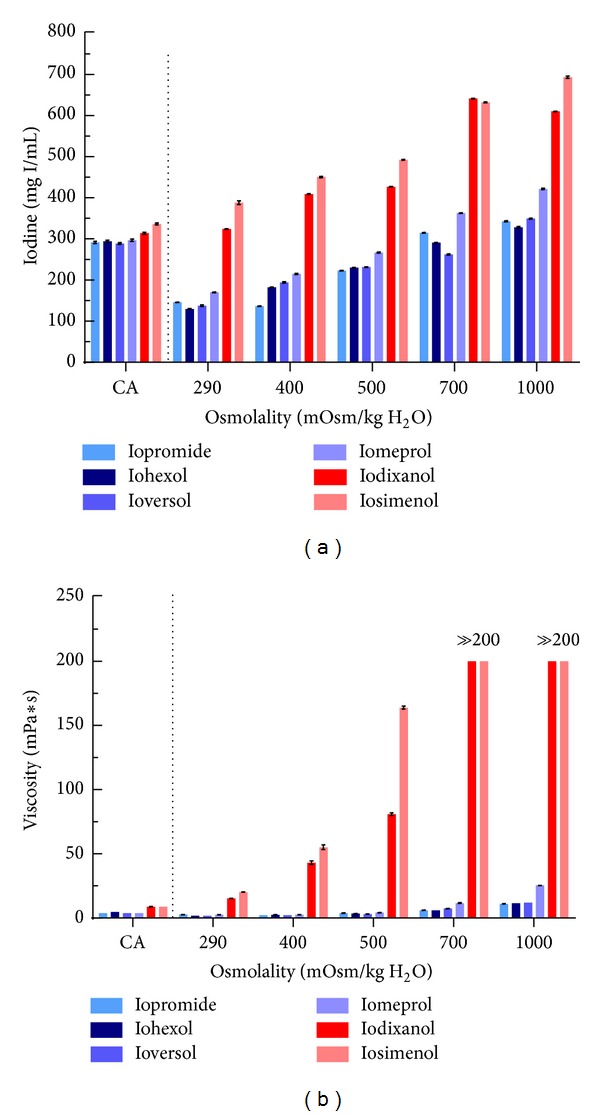
Changes of concentration and viscosity of solutions of six different contrast media (four LOCM depicted in blue: iopromide 300, iohexol 300, ioversol 300, and iomeprol 300; two IOCM depicted in red: iodixanol 320 and iosimenol 350) caused by *in vitro* dialysis to emulate the renal tubular concentration process. Iodine concentration (a) and viscosity (b) of the respective contrast agent solutions as marketed/formulated for clinical use (marked as CA) and after dialysis with PEG solutions with osmolalities of 290, 400, 500, 700, and 1000 mosm/kg H_2_O. Data are mean ± SEM. Please note that the viscosity of both IOCM solutions after the dialysis at 700 and 1000 mosm/kg H_2_O was so high that it even exceeded the upper measurement limit of the viscometer. Redrawn from data in [34].

The quantitative effects of osmotic forces comparable to those present in the different renal layers on water reabsorption and, thus, on the concentrating of CM have recently been illustrated by an *in vitro* dialysis study (Figure 2(a)) [34]. Six CM solutions as marketed/formulated for clinical use were studied: four LOCM and two IOCM; with comparable iodine concentrations, the viscosities of the IOCM solutions are about twice those of LOCM solutions. Dialysis of the solutions at ambient osmotic pressure of 290 mosmol/kg H_2_O (i.e., isoosmotic to plasma) resulted in a decrease in the iodine concentration of LOCM but not IOCM solutions, as can be expected from the net water inflow into the solutions that is driven by the LOCM solutions' higher osmolalities. With increasing ambient osmotic pressures, water is progressively extracted from the solutions such that the iodine concentrations progressively increase. The iodine concentrations of IOCM exceeded those of LOCM on each osmotic pressure step. At ambient osmotic pressures of 1000 mosmol/kg H_2_O (i.e., comparable to the inner medulla), iodine concentrations of LOCM solutions were somewhat higher than in the marketed solutions, yet iodine concentrations of IOCM solutions were doubled as compared to their marketed solutions. Owing to the exponential concentration-viscosity relationship, this was accompanied by an increase in the viscosity of LOCM solutions, yet the viscosity increase of IOCM solutions was several times larger; in fact, it by far exceeded the measuring range of the used viscometer (Figure 2(b)) [34].

## 5. Why the Hydration Status Impacts on Tubular Fluid Viscosity

In line with these *in vitro* observations, *in vivo* studies that directly compared urine viscosities following LOCM versus IOCM administration in dogs and rats clearly demonstrated larger increases in urine viscosities following IOCM [34, 39–46]. This was also confirmed by a small series of patients [44]. In all these studies, the subjects were well hydrated and presumably euvolaemic due to ample administration of fluids. It does not therefore surprise that, in absolute terms, the increases in urine viscosity were rather small. The degree of tubular water reabsorption depends on a subject's hydration and volume status (Figure 1). In subjects that are not well hydrated and/or hypovolaemic, physiological mechanisms that aim at water and/or volume preservation are triggered, in particular, activation of the renin-angiotensin system and of vasopressin [47–49]. Angiotensin II and vasopressin augment tubular fluid reabsorption, which further increases the tubular concentration of CM and, due to the concentration-viscosity relationship, overproportionally increases urine viscosity. Accordingly, dehydration and/or volume contraction are major individual risk factors for CIAKI; hence the strong recommendation of prehydration that is embodied in clinical guidelines [8, 9].

It must be noted that terms such as “volume status,” “well hydrated,” and “euvolaemia” are generally used in a qualitative rather than a quantitative sense. Moreover, terms like “hydration,” “dehydrated,” and so forth are used throughout the literature on CIAKI including current guidelines without a clear distinction whether water or volume (isotonic fluid) is meant [1–9]. For instance, the term “dehydration,” in its strict sense, denotes a deficit of water with an ensuing increase in osmolality (hypertonic dehydration) but is often used as a synonym for a deficit in isotonic volume (volume contraction). The imprecise use of these terms may reflect the fact that a quantitative assessment of a patient's volume status is not easily achieved in clinical practice, particularly in outpatients and, as long as the patient does not display clinical signs of a marked volume deficit, is seldom performed. In addition, large portions of patients who undergo CM-related interventions are at old age and therefore prune to suffer from (hypertonic) dehydration due to impaired sensation of thirst [2, 50]. Current guidelines recommend “hydration” either by isotonic NaCl or sodium bicarbonate, by hypotonic 0.45% NaCl solution or by water per os, without requiring prior quantitative assessment of the patient's hydration and volume status [8, 9]. However, irrespective of whether hydration is achieved by isotonic or hypotonic fluid, or just by water per os, the patients' kidneys will benefit from increased tubular flow.

**Figure 3:**
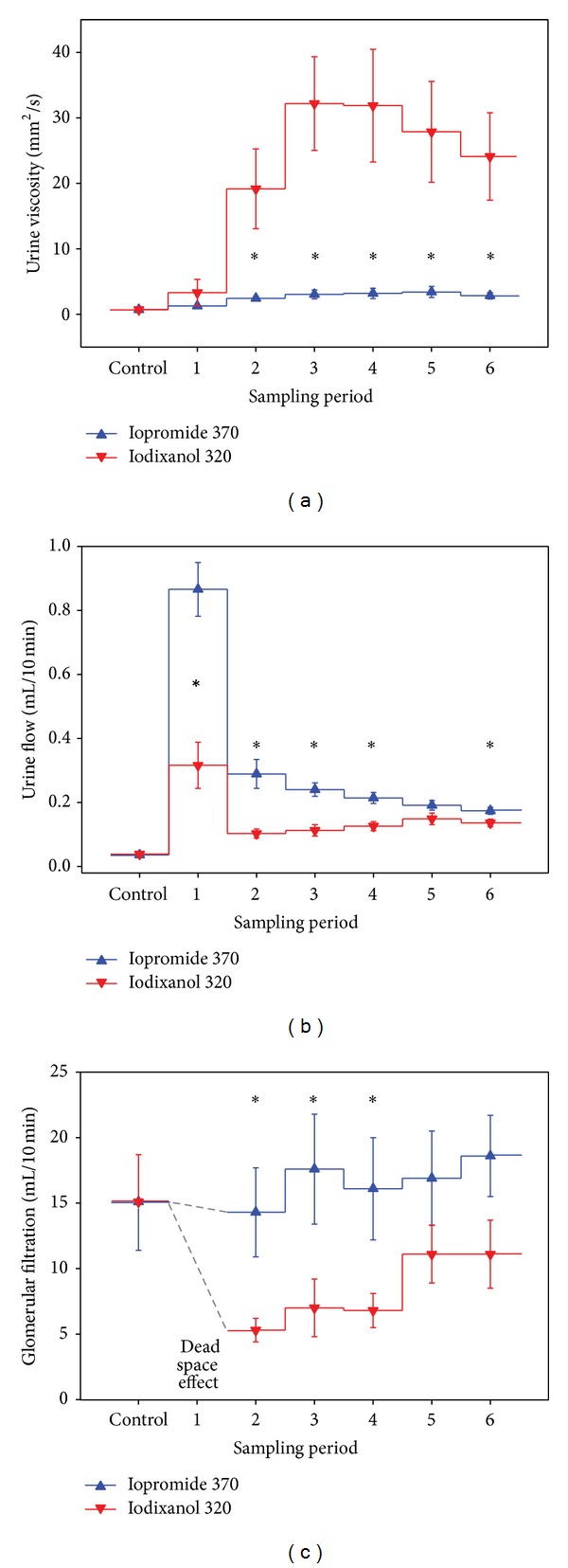
Viscosity of urine samples, urine flow rate, and glomerular filtration rate (GFR; measured by creatinine clearance) in rats before (control) and following contrast media administration (six 10 min sampling periods). Iopromide 370 mg I/per mL or iodixanol 320  mg I/mL was injected into the thoracic aorta as a bolus of 1.5 mL. Rats had access to drinking water prior to the experiment but were not hydrated by infusions. Data are mean ± SEM. *P < 0.05 iopromide versus iodixanol. In all sample periods after contrast media injection, urine viscosities and urine flow rates were significantly higher than in the respective control sample. In rats receiving iodixanol, GFR was significantly lower than control GFR 10 to 40 min after iodixanol injection, whereas GFR remained unchanged in rats receiving iopromide. Note that GFR values for the first period following contrast media injection are not depicted, as high creatinine clearance values obtained for this period do not represent actual increases in GFR but rely on the dead-space effect. Redrawn from data in [39].

Despite the guidelines' strong recommendations, a considerable portion of patients in everyday clinical practice is, for various reasons, not sufficiently hydrated [24, 51]. For ethical reasons, prospective clinical trials can, of course, only be performed according to protocols that include ample fluid administration. In order to fill in the gap in knowledge, freely drinking rats were studied that concentrated their urine to an extent comparable to nonhydrated humans [39]. As shown by Figure 3(a), injection of the IOCM, iodixanol 320 mg I/mL, into the thoracic aorta led to a massive increase in urine viscosity, whereas following the LOCM, iopromide 370 mg I/mL, urine viscosity was only moderately elevated. Micropuncture studies in rats and functional MRI studies in rats also found tubular fluid viscosity to be much higher following IOCM than LOCM [43, 52].

## 6. Why CM Osmolality Impacts on Tubular Fluid Viscosity

The large difference observed between urine viscosities following iodixanol versus iopromide (Figure 3(a)) cannot be explained by the viscosity of the CM solutions alone, for which the difference is much less (~10 versus ~7 mm^2^/s). Here, the difference in CM osmolalities plays a major role. The more nonreabsorbable osmolytes the tubular fluid contains the smaller is the osmotic gradient between the tubules' lumen and the interstitium that drives water reabsoption. Therefore, nonreabsorbable CM all induce osmotic diuresis, but to different degrees: the higher the osmolality of a given CM the stronger is its diuretic effect (Figure 1). Thus, the LOCM, iopromide, generates much more diuresis than the IOCM, iodixanol, and does, thereby, prevent a major increase in urine viscosity even in animals that are not well hydrated (Figure 3(b)) [39].

The tubular osmotic force of iopromide is much greater than that of iodixanol for two reasons. First, the osmolality of the iopromide 370 mg I/mL solution is more than twice as high as that of the iodixanol 320 mg I/mL solution (770 versus 290 mosmol/kg H_2_O). Second, because solutions of pure iodixanol drug substance with 320 mg I/mL are hypo-osmolar (210 mosmol/kg H_2_O), reabsorbable osmolytes are added to the marketed solution to render it isoosmolar [40]. As a portion of these osmolytes will be reabsorbed, the tubular osmotic force of iodixanol is further reduced. In accordance, in another study in rats, a solution of the pure iodixanol drug substance (320 mg I/mL) induced less diuresis than the marketed solution [40]. Moreover, in this study, the effect of increasing the osmolality of the marketed iodixanol 320 mg I/mL solution by nonreabsorbable mannitol was tested. Increasing the osmolality of iodixanol solution to 610 mosmol/kg H_2_O indeed enhanced diuresis and, thereby, decreased urine viscosity [40]. The finding that LOCM, by virtue of their higher osmolality, induce larger diuresis than IOCM has been confirmed by several preclinical studies including studies in very well-hydrated rats [34, 39, 43–46]. Taken together, the higher osmolality of LOCM as compared to IOCM bears the advantage of preventing excessive urine viscosity levels.

## 7. Why High Tubular Fluid Viscosity Conveys Deleterious Effects

The fluid flow rate through a tube increases with the pressure gradient and decreases with the flow resistance. The resistance increases proportionally to fluid viscosity; it also increases with the tube's length and decreases with its radius (Poiseuille's law). Thus, any increase in fluid viscosity will reduce the flow rate at a given pressure gradient. The ensuing congestion, in turn, will increase the upstream pressure. Considering the minute diameter and the relatively great length of renal tubules, it does not surprise that CM-induced high tubular fluid viscosity increases tubular pressure and may hinder glomerular filtration. In fact, early micropuncture studies in rats found that the IOCM, iotrolan, increased tubular pressure much more and decreased single nephron GFR much more as compared to the HOCM and LOCM studied [53, 54]. As shown by Figure 3(c), following injection of the IOCM, iodixanol, but not the LOCM, iopromide, into the thoracic aorta of rats, a marked transient decrease in GFR is observed that parallels the increase in urine viscosity [39]. As expected, prehydration by saline or bicarbonate infusions blunted the increase in tubular viscosity and, thereby, alleviated the iodixanol-induced decreases in GFR but did not prevent it [39, 45].

**Figure 4:**
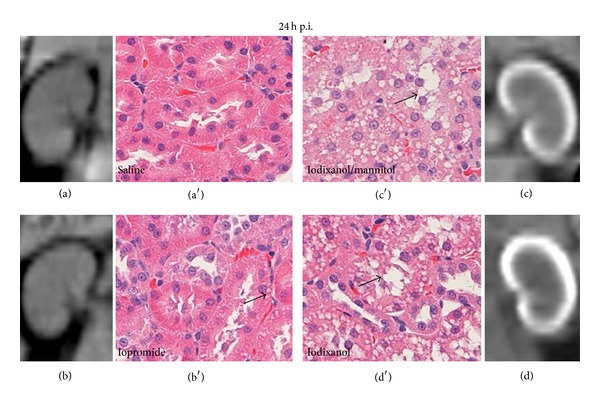
Exemplary computed tomographic (CT) scans to assess renal iodine retention and exemplary histological images (hematoxylin-eosin staining) to assess formation of vacuoles in proximal tubular cells, both taken 24 hours after injection (24 h p.i.) of either saline (a), marketed iopromide 300 mg I/mL solution (b), iodixanol 320 mg I/mL solution with mannitol added to elevate the solution's osmolality (c), or marketed iodixanol 320 mg I/mL solution (d). CM were administered intravenously at a dose of 4 g I/kg of body mass. CT scans show predominantly cortical iodine retention 24 h p.i. for the marketed iodixanol solution, less retention following the iodixanol/mannitol solution, and virtually none following iopromide and saline. Formation of vacuoles (arrows) in proximal tubular cells was prominent 24 h p.i. for the marketed iodixanol solution, slightly less following the iodixanol/mannitol solution and sparse after iopromide and saline. Reprinted from [40].

It is possible that mechanisms other than viscosity-related increase in tubular pressure may contribute to lower GFR: in a recent *in vitro* study in isolated mouse afferent and efferent glomerular arterioles perfused with electrolyte solutions that contained diluted iodixanol, a small but significant vasoconstriction of afferent arterioles was found [55]. As will be discussed below, reduced blood perfusion and tissue oxygenation, in particular, medullary hypoperfusion and hypoxia, are pivotal pathophysiological elements in CIAKI. Because afferent vasoconstriction does not only reduce perfusion and GFR but at the same time also decreases oxygen-dependent tubular reabsorption, it is unclear whether or not it contributes to renal injury. In any case it must be noted that a decrease in GFR following CM administration, despite being the basis of clinical diagnosis, is *per se* not proof of renal injury. The viscosity-induced increase in tubular pressure, however, may well contribute to medullary hypoperfusion and hypoxia: in the face of the rather tough renal capsule, circular distension of the tubules must result in compression of medullary vessels such as the vasa recta [53, 56, 57]. Another effect of increased tubular fluid viscosity is that increased flow resistance markedly slows tubular flow [44], so that the intrarenal retention time of IOCM is much longer than that of LOCM, as shown in rats and minipigs [35, 58, 59]. In the kidneys of renally impaired ZSF1 rats, IOCM were even retained for 2 weeks [35]. Recent comparisons in healthy rats among the marketed iopromide 300 mg I/mL solution, the marketed iodixanol 320 mg I/mL solution and the above-mentioned mannitol-iodixanol 320 mg I/mL (610 mosmol/kg H_2_O) demonstrated that the higher the viscosity and the lower the osmolality, the longer is the intrarenal CM retention (Figure 4) [40, 58]. As has been recognised in the 1990s already [60], prolongation of retention is in part related to the induction of vacuoles in proximal tubular cells (Figure 4) [40, 58]. Probably the worst effect of the viscosity-related intrarenal retention is the prolonged exposure of tubular epithelial cells to potentially cytotoxic CM.

**Figure 5:**
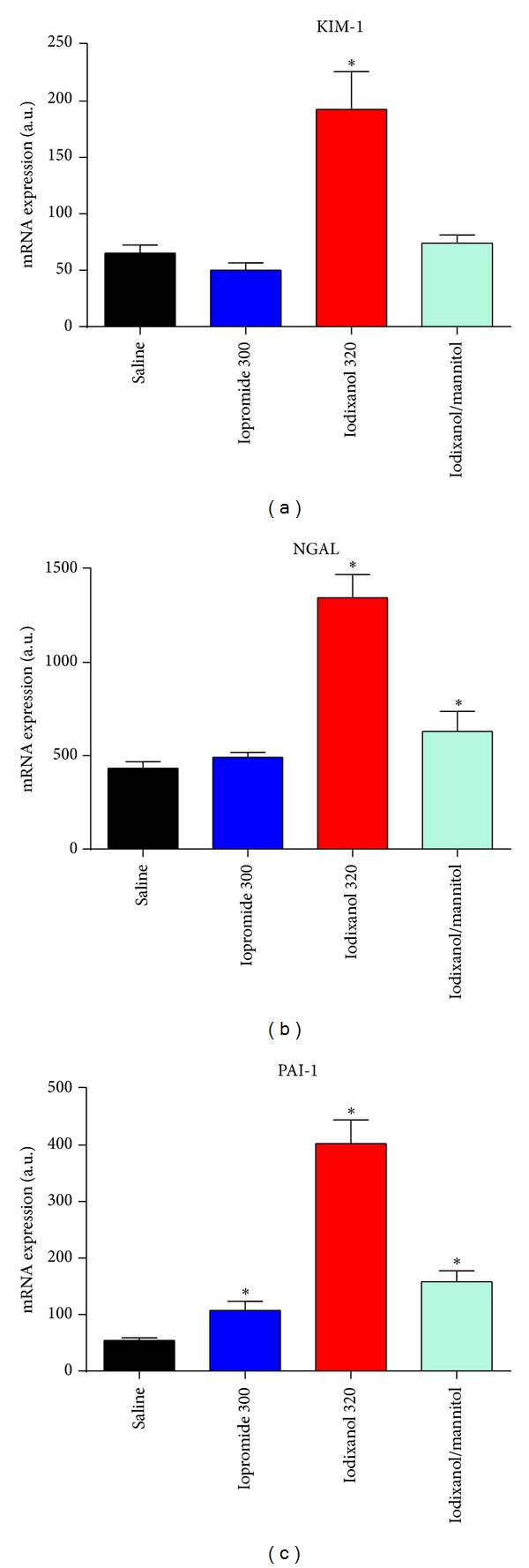
Renal tissue expression (mRNA levels as analysed by real-time PCR) of kidney injury molecule 1 (KIM-1) and of neutrophil gelatinase associated lipocalin (NGAL) as quantified 24 hours after injection and that of plasminogen activator inhibitor-1 (PAI-1) as quantified 2 hours after injection of either saline, marketed iopromide 300 mg I/mL solution, marketed iodixanol 320 mg I/mL solution, or iodixanol 320 mg I/mL solution with mannitol added to elevate the solution's osmolality. CM were administered intravenously at a dose 4 g I/kg of body mass. Data are mean ± SEM. *P < 0.05 versus saline. Data taken from [35, 40, 58].

**Figure 6:**
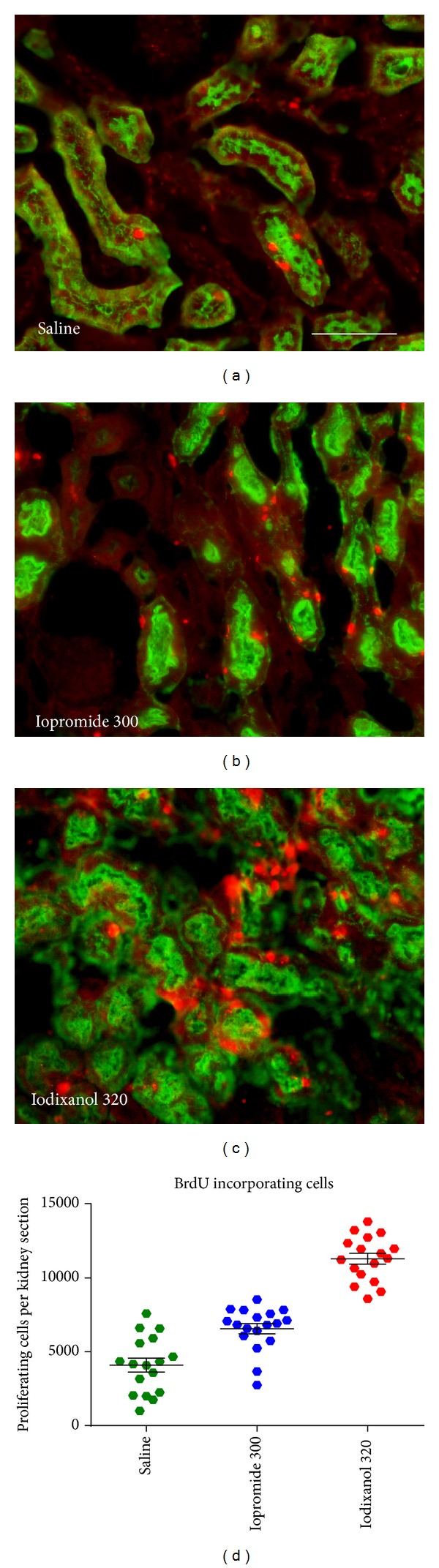
Elevated proliferation rate of renal cells following CM administration as assessed by immunofluorescence analysis of the incorporation of bromdesoxyuridine (BrdU; red staining) into proximal tubular cells (indicated by green staining of aquaporin-1; scale bar 100 *μ*m). The BrdU incorporation time was 46 hours, starting 2 h after i.v. injection of either saline (a), iopromide 300 mg I/mL (b), or iodixanol 320 mg I/mL solution (c); CM doses were 4 g I/kg of body mass. Semiquantitative analysis of the BrdU-incorporated cells (d). Reprinted from [58].

*In vitro* studies clearly demonstrated that CM of all classes exert cytotoxic effects: cultured cells of various types including renal tubular epithelial and vascular endothelial cells present signs of cell damage, oxidative stress, and/or apoptosis when exposed to CM [61–63]. The cytotoxicity of CM may rely on iodine that can be released from CM by photolysis [38]. Minute amounts of free iodine may be highly cytotoxic [64]. Recent *in vivo* studies in rats assessed renal injury following i.v. CM administration by studying renal tissue expression of several AKI biomarkers using real-time PCR (Figure 5) [35, 40, 58]. Expressions of kidney injury molecule 1 (KIM-1, a marker of tubular injury, mainly proximal cells) [65] and of neutrophil gelatinase associated lipocalin (NGAL, here a marker of tubular injury, mainly distal cells) [66, 67] were quantified 24 hours after CM injection and those of plasminogen activator inhibitor-1 (PAI-1, here mainly a marker of injury to collecting duct cells) [68] 2 hours after CM injection. Whereas administration of iopromide 300 mg I/mL did not change renal KIM-1 and NGAL expressions, iodixanol 320 mg I/mL resulted in ~3fold upregulation of NGAL and KIM-1. Iopromide induced a ~2fold upregulation and iodixanol a ~7.4-fold upregulation in PAI-1. Injection of the above-mentioned iodixanol solution with mannitol-induced elevation of osmolality markedly reduced the effects on these AKI markers as compared to the marketed iodixanol solution. These results mirror the different retention times: the higher the viscosity and the lower the osmolality, the longer are the cells exposed to CM and the more they are injured. The effect of viscosity-induced renal injury was further demonstrated by histological immunofluorescence analysis of the incorporation of bromdesoxyuridine into cells (BrdU, a marker of cell proliferation following injury) [69] 48 hours after CM injection (Figure 6) [58]. The number of proliferating cells increased significantly more following iodixanol versus iopromide.

## 8. Why High CM Viscosity Conveys Medullary Hypoperfusion and Hypoxia

Hypoxia in the renal medulla is a hallmark of CIAKI [1, 7, 33, 70–73]. Medullary hypoxia is part of a vicious circle that entails cellular damage, oxidative stress, and vasoconstriction. The outer medulla is especially vulnerable to hypoxia: oxygen requirements are high due to salt reabsorption in Henle's thick ascending limbs, while oxygen delivery is sparse. Oxygen supply to the medulla is low because of the small medullary fraction of total renal blood flow (<10%) and of arteriovenous oxygen shunt diffusion. CM in the medulla affect the fragile balance between oxygen delivery and consumption by several mechanisms, the main mechanism being reduced blood perfusion [7, 33, 70–72]. Constriction of preglomerular vessels, although possibly contributing to the CM-induced drop in GFR, has small if any impact on medullary oxygenation: blood flow reduction in medullary vessels that emerge from efferent arterioles is usually accompanied by reduced solute reabsorption and, thus, reduced oxygen consumption. Medullar hypoxia is therefore mainly caused by CM effects on the medullary vessels themselves, in particular, on the long and narrow vasa recta.

At least three CM effects, possibly acting in concert, contribute to the increase in medullary vascular resistance. First, as mentioned above, viscosity-induced increase in tubular pressure probably compresses the vasa recta [53, 56]. Second, CM can constrict descending vasa recta (DVR), as DVR are lined by pericytes that are able to contract [74]. In a series of *in vitro* studies in isolated DVR obtained from rats and human beings, the effects of CM on vascular diameter and reactivity and on endothelial generation of nitric oxide (NO) and reactive oxygen species (ROS) were tested [75–77]. In these studies, DVR were perfused by crystalloid solutions that contained CM in low concentrations so that the solutions' osmolalities and viscosities equaled those of plasma. CM of all classes caused similar degrees of DVR constriction. In addition, the vasoconstrictive response to angiotensin II was enhanced by CM. The concentration of ROS was increased, which may rely on CM-induced damage of endothelial cells [77, 78]. Endothelial injury may also account for the impaired NO production by DVR observed in these studies [75]. Under *in vivo* conditions, vasomotion of DVR is subject to tubulovascular crosstalk [74]. It is thus most probable that signals from injured tubular epithelium contribute to DVR vasoconstriction. On the other hand, the impaired endothelial NO generation of DVR could, at least in part, be compensated for *in vivo* by hemoglobin-generated NO: hemoglobin reduces nitrite to NO in hypoxic areas such that NO and, thus, vasodilation are provided “on demand” [79, 80].

The third CM effect that contributes to medullary hypoperfusion and hypoxia again relies on viscosity, namely, on increased blood viscosity. This was demonstrated by a study in rats that compared four solutions: the IOCM, iodixanol, the LOCM, iopromide (both at 320 mg I/mL), mannitol solution with equal osmolality as iopromide, and dextran 500,000 solution with equal viscosity as iodixanol [44]. In this study, the solutions were injected into the thoracic aorta and local tissue perfusion and oxygen tension (pO_2_) was monitored by invasive techniques, namely, laser Doppler probes and fluorescence-quenching optodes. Only the high viscous solutions (iodixanol and dextran) resulted in long lasting medullary hypoperfusion and, thus, in lower medullary pO_2_. Because dextran 500,000 is not filtered in the glomeruli, the medullary hypoperfusion induced by this high viscous solution cannot rely on high tubular fluid viscosity. However, events corresponding to the tubular concentration process take place in the DVR: as blood flows through the hypertonic environment of the medulla, a portion of plasma water will leave these vessels towards the hypertonic interstitium. This will enrich CM within the vessels, thus increasing blood viscosity. Both of the high osmolar solutions mannitol and the LOCM, iopromide, did not affect medullary perfusion and pO_2_.

A recent study in rats that utilised the same monitoring techniques corroborated the marked and prolonged (observation time 60 min after CM) medullary hypoperfusion and hypoxia following bolus injection of the IOCM, iodixanol, into the thoracic aorta [80]. Another study that assessed tissue perfusion by laser Doppler probes in rats directly compared the effects of i.v. injection of the LOCM, ioxaglate, with that of the IOCM, iodixanol: only iodixanol resulted in significant medullary hypoperfusion [81]. A further study in rats compared the effects of i.v. injection of the HOCM, iothalamate, with the IOCM, iotrolan, and found that outer medullary hypoperfusion was pronounced by iotrolan versus iothalamate [82]. In a study in dogs, either the LOCM, ioxaglate, or the IOCM, iodixanol, were injected into the renal artery and local tissue perfusion and pO_2_ were monitored by laser Doppler probe and Clark-type electrodes, respectively: both CM reduced medullary perfusion and pO_2_, yet hypoperfusion and hypoxia lasted significantly longer following iodixanol than ioxaglate [42]. Using Clark-type electrodes in rats, the IOCM, iotrolan, was found to decrease medullary pO_2_ much more than the LOCM, iopromide [83].

**Figure 7:**
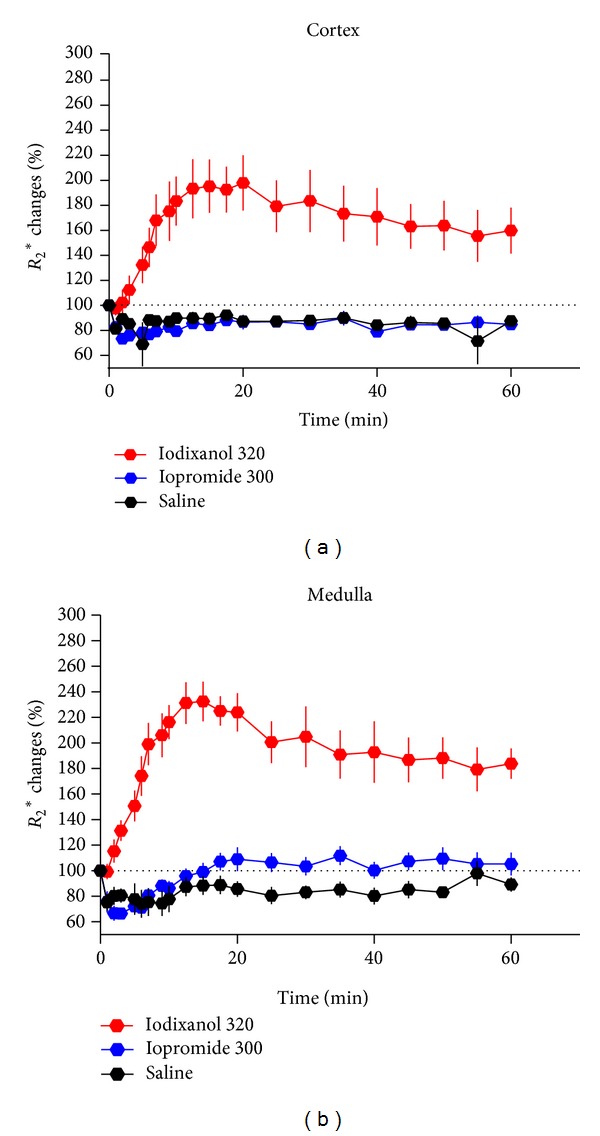
Time course of alterations in renal oxygenation as estimated by blood oxygen level-dependent (BOLD) MRI for the cortex and the medulla depicted as percentage changes (mean ± SEM) in *R*_2_* from baseline (100%, dotted lines). Increase in *R*_2_* above baseline signifies reduced (blood) oxygenation, that is, hypoxia. Intravenous injection of either saline, iopromide 300 mg I/mL, or iodixanol 320 mg I/mL was done at time 0; CM doses were 4 g I/kg of body mass. Data taken from [40].

The invasive pO_2_-probes provide calibrated quantitative measurements but cover only small tissue areas, which is not optimal, because renal oxygenation displays considerable spatial heterogeneity [84–86]. Magnetic resonance imaging (MRI) enables spatially resolved monitoring, and blood oxygen level-dependent (BOLD) MRI is increasingly used to assess changes in kidney oxygenation as induced by various interventions [85]. A recent BOLD study in rats showed a brief transient increase followed by a decrease in inner and outer medullary oxygenation upon bolus injection of the IOCM, iodixanol, into the thoracic aorta [56]. A study in rabbits found oxygenation in the outer stripe of the outer medulla unchanged but that in the inner stripe decreased following i.v. injection of the LOCM, iopamidol [87]. Direct comparisons between BOLD studies are impossible because BOLD is not quantitatively calibrated and the specific MR protocols differ considerably among studies [85, 86]. However, using a cross-over design to enable direct intraindividual comparison, the effects of i.v. injection of the LOCM, iopromide, versus the IOCM, iodixanol, were studied in pigs. This BOLD study indicates that iodixanol impairs inner medullary but not outer medullary oxygenation, while iopromide did not impair oxygenation in both layers [88]. A recent study in rats also compared oxygenation changes following iopromide versus iodixanol and found both medullary and cortical oxygenation decreased upon i.v. injection of iodixanol but not iopromide (Figure 7) [40]. Finally, a study in rats compared the effects of high viscosity versus high osmolality by use of the HOCM, iothalamate, and the IOCM, iodixanol [89]. Iodixanol dose dependently impaired medullary oxygenation, while iothalamate did not. In order to emulate endothelial dysfunction, which is an important individual risk factor for CIAKI, another group of rats was exposed to inhibition of nitric oxide and prostaglandin before administration of CM. This pretreatment aggravated the decrease in medullary oxygenation following iodixanol but also rendered the rats receiving iothalamate susceptible for impaired medullary oxygenation [89].

Taken together, the bulk of evidence from animal studies as presented here clearly indicates that LOCM are superior to IOCM when it comes to renal adverse effects. This is due to both the lower viscosity and the higher osmolality of LOCM. The latter also becomes obvious from the results obtained with iodixanol solutions in which the osmolality was increased by addition of mannitol. Considering these clear-cut results, two questions immediately come to mind.

## 9. Why Excessive CM Osmolality May Convey Deleterious Effects

When high CM osmolality indeed conveys beneficial effects, why pioneer HOCM (osmolalities ~1000–2500 mosmol/kg H_2_O) were then associated with a greater CIAKI incidence in at-risk patients than LOCM (osmolalities: ~400–800 mosmol/kg H_2_O)? Here, quantitative aspects of hyper-osmolality play a pivotal role. Adding a hyperosmolar solution to normal tissue, of course, results in cell shrinkage, yet the cells of the area at risk for CIAKI, the outer medulla, are constantly exposed to osmolalities of ~400–600 mosmol/kg H_2_O, and the cells of the inner medulla to osmolalities up to ~1200 mosmol/kg H_2_O. Direct hyperosmolar injury of cells can occur only if tubular fluid osmolality is in excess of ambient medullary osmolality. Interestingly, a correlation between CM osmolality and nephrotoxicity was observed for CM with osmolalities >800 mosmol/kg H_2_O [1, 32]. It is thus possible that HOCM solutions >800 mosmol/kg H_2_O become concentrated in tubules to such an excessive extent. However, to our knowledge, this has never been shown in human beings.

A number of additional explanations have been forwarded for the higher CIAKI incidence of HOCM versus LOCM. Because HOCM and other high-osmolar solutions can cause a distinct histological pattern with vacuolization of tubular cells (osmotic nephrosis) these alterations were thought to rely on osmotic forces. This explanation proved wrong: the alterations are caused by pinocytosis, and vacuolization was found even pronounced upon IOCM [40, 60, 90, 91]. Increased osmotic workload with ensuing increase in tubular oxygen consumption may contribute to HOCM-induced medullary hypoxia: furosemide that reduces oxygen-dependent tubular transport alleviated iothalamate-induced medullary hypoxia in rats [92]. This mechanism does not seem to play a role in LOCM: furosemide did not improve medullary pO_2_ upon iopromide injection [93]. HOCM influence the shape and rigidity of erythrocytes, making it more difficult for them to flow through the narrow DVR, which could also contribute to medullary hypoperfusion [94]. A video-microscopy study indicated that HOCM, but also LOCM and IOCM, may induce the so-called sludge effects in vasa recta [95]. Finally, it must be noted that several studies indicate that adverse effects of HOCM may rely on their electric charge rather than their high osmolality [96].

## 10. How Preclinical Results Compare to That of Clinical Studies

In the light of the evidence from preclinical studies it may seem surprising that current meta-analyses of up to 36 prospective randomised controlled clinical trials conclude that there is no significant difference in CIAKI incidence between LOCM and IOCM [97–99]. Apart from the heterogeneity of the trials included in the meta-analyses and the poor sensitivity of SCrea (the end point used in the vast majority of trials), there is a most probable explanation: virtually all prospective clinical trials are performed according to protocols with ample fluid administration. Because of the exponential concentration-viscosity relationship, even minor dilution will greatly reduce tubular fluid viscosity. The undesirable effects of high tubular fluid viscosity are likely to be seen only in nonhydrated patients.

In contrast to prospective clinical trials, in everyday clinical practice, many patients are not sufficiently hydrated [51]. Such patients are certainly among the 57,925 patients included in a retrospective registry study on cardiac interventions [100]. In this study, patients who received the IOCM, iodixanol, experienced clinically relevant renal failure including requirement of dialysis two to three times as often as patients who received LOCM. Likewise, another registry study in 58,957 patients found CIAKI incidence significantly higher following the IOCM, iodixanol, versus LOCM (as assessed by SCrea, by required dialysis, and a higher in-hospital mortality) [101]. However, in this latter study, iodixanol was used more frequently in older patients with more comorbidities and worse pre-CM renal function, and in propensity-matched models, the differences did not reach statistical significance. It is conceivable that patients with higher risk scores received better hydration; unfortunately, the authors were unable to assess differences in fluid administration [101]. Taken together, in patients who are not sufficiently hydrated, LOCM probably have an advantage over IOCM.

## 11. Conclusions

This review of preclinical studies clearly indicates that the renal safety of modern CM varies dramatically with regard to their osmolality and viscosity. High CM viscosity is a key element in the pathophysiology of CIAKI, because the hyperosmolar environment of the renal medulla results in CM enrichment in both the tubules and the vasculature. Thus, fluid viscosity increases exponentially and flow through medullary tubules and vessels decreases. Reducing the flow increases the contact time of cytotoxic CM with the tubular epithelium and vascular endothelium. This triggers a vicious circle of cell injury, medullary vasoconstriction, and hypoxia. Moreover, glomerular filtration declines due to congestion of highly viscous tubular fluid. The viscosities of both LOCM and IOCM markedly exceed those of plasma, yet the higher osmolalities of LOCM convey a renoprotective effect: by virtue of their greater tubular osmotic force, LOCM cannot be concentrated to the same extent as IOCM. In consequence, tubular fluid viscosity may not exceed a critical level. It must be pointed out, however, that the preclinical studies reported here were performed in healthy animals. In contrast, clinical examinations and interventions that make use of intravascularly applied CM, and, thus, also clinical trials on CIAKI, of course, include patients who suffer from various comorbidities so that none of today's CM is without potential clinical nephrotoxicity [102].
